# S-Nitrosoglutathione (GSNO)-Mediated Lead Detoxification in Soybean through the Regulation of ROS and Metal-Related Transcripts

**DOI:** 10.3390/ijms24129901

**Published:** 2023-06-08

**Authors:** Nusrat Jahan Methela, Mohammad Shafiqul Islam, Da-Sol Lee, Byung-Wook Yun, Bong-Gyu Mun

**Affiliations:** 1 Department of Plant Biosciences, School of Applied Biosciences, College of Agriculture & Life Science, Kyungpook National University, Daegu 41566, Republic of Korea; 2 Department of Agriculture, Faculty of Science, Noakhali Science and Technology University, Noakhali 3814, Bangladesh

**Keywords:** nitric oxide, S-nitrosoglutathione (GSNO), reactive oxygen species, metal stress, stress tolerance

## Abstract

Heavy metal toxicity, including lead (Pb) toxicity, is increasing in soils, and heavy metals are considered to be toxic in small amounts. Pb contamination is mainly caused by industrialization (e.g., smelting and mining), agricultural practices (e.g., sewage sludge and pests), and urban practices (e.g., lead paint). An excessive concentration of Pb can seriously damage and threaten crop growth. Furthermore, Pb adversely affects plant growth and development by affecting the photosystem, cell membrane integrity, and excessive production of reactive oxygen species (ROS) such as hydrogen peroxide (H_2_O_2_) and superoxide (O_2_^−^). Nitric oxide (NO) is produced via enzymatic and non-enzymatic antioxidants to scavenge ROS and lipid peroxidation substrates to protect cells from oxidative damage. Thus, NO improves ion homeostasis and confers resistance to metal stress. In the present study, we investigated the effect of exogenously applied NO and S-nitrosoglutathione in soybean plants Our results demonstrated that exogenously applied NO aids in better growth under lead stress due to its ability in sensing, signaling, and stress tolerance in plants under heavy metal stress along with lead stress. In addition, our results showed that S-nitrosoglutathione (GSNO) has a positive effect on soybean seedling growth under lead-induced toxicity and that NO supplementation helps to reduce chlorophyll maturation and relative water content in leaves and roots following strong bursts under lead stress. GSNO supplementation (200 µM and 100 µM) reduced compaction and approximated the oxidative damage of MDA, proline, and H_2_O_2_. Moreover, under plant stress, GSNO application was found to relieve the oxidative damage by reactive oxygen species (ROS) scavenging. Additionally, modulation of NO and phytochelatins (PCS) after prolonged metal reversing GSNO application confirmed detoxification of ROS induced by the toxic metal lead in soybean. In summary, the detoxification of ROS caused by toxic metal concentrations in soybean is confirmed by using NO, PCS, and traditionally sustained concentrations of metal reversing GSNO application.

## 1. Introduction

In recent years, heavy metal toxicity in soil has been an issue of concern worldwide. Particularly, cadmium (Cd), lead (Pb), mercury (Hg), chromium (Cr), and arsenic (As) are the most problematic heavy metals, considering their high toxicity in soil [1]. Heavy metals, such as Pb, are found in soil and are toxic even in trace amounts. Pb contamination primarily occurs by industrialization (e.g., smelting and mining), agricultural practices (e.g., sewage sludge and pests), and urban practices (e.g., lead paints) [2]. Pb is non-biodegradable and can move from soil into water and eventually enter crops. Furthermore, excessive lead can cause serious damage and pose a threat to crop growth [3,4,5]. Notably, Pb can adversely affect plant growth and development by reducing nutrient uptake and severely affecting photosystems, nitrogen metabolism, cell membrane integrity, chlorosis, and necrosis. However, plants possess their own defense system to mitigate ultimate toxicity caused by adverse environmental stresses [6,7]. Overproduction of reactive oxygen species (ROS) such as hydrogen peroxide (H_2_O_2_), superoxide (O_2_^.−^), and singlet oxygen (^1^O_2_) is a typical response of plants under heavy metal exposure. Several studies have reported Pb-induced ROS production in plants. In *Zea mays*, upregulation of the antioxidant system prevented the overproduction of ROS induced by Pb. [8]. Similarly, antioxidative activities such as catalase (CAT), ascorbate peroxidase (APX), and dehydroascorbate reductase (DHAR) were increased in response to different levels of Pb exposure in *Sedum alfredii* [9]. However, plants can further boost their antioxidants to scavenge ROS with the exogenous supplementation of growth regulators such as nitric oxide (NO), which can improve crop quality and yield. NO has been identified in the sequestration of metal toxicity against Cr [10], Pb [11,12], Cd [13], vanadium (V) [14], and As [15]. NO can increase the activity of both enzymatic and non-enzymatic antioxidants to scavenge ROS and lipid peroxidative substrates, and therefore, protect cells from oxidative damage against heavy metal stress by reducing their uptake accumulation, and translocation. Consequently, NO improves ionic homeostasis and provides tolerance to metal stress [16]. Moreover, NO is a signaling and highly reactive biomolecule that has the ability to alter the structure and activity of proteins [12]. In addition, depending on the type of donor and the concentration when supplied exogenously, NO can function as a beneficial or harmful molecule in plants. S-nitrosoglutathione (GSNO) is an S-nitrosylated derivative of cellular thiol glutathione (GSH) and is considered to be one of the most important NO donors [16]. GSNO has been found to have a positive affect for long periods as a NO source against water stress in sugarcane [17]. However, there are limited reports of GSNO application in plants. To the best of our knowledge, S-nitrosothiols are an emerging source of NO donors because of their potential applications. In the present study, we investigated the potential function of GSNO for controlling lead accumulation, plant development, and antioxidative activities in GSNO-treated soybean plants under Pb stress Furthermore, we also investigated the influence of GSNO on gene expression related to NO, phytochelation, and heavy metal transportation to alleviate lead-induced toxicity.

## 2. Results

### 2.1. Nitric Oxide Enhances Plant Growth, Chlorophyll, and Relative Water Content under Lead-Induced Toxicity

**Figure 1:**
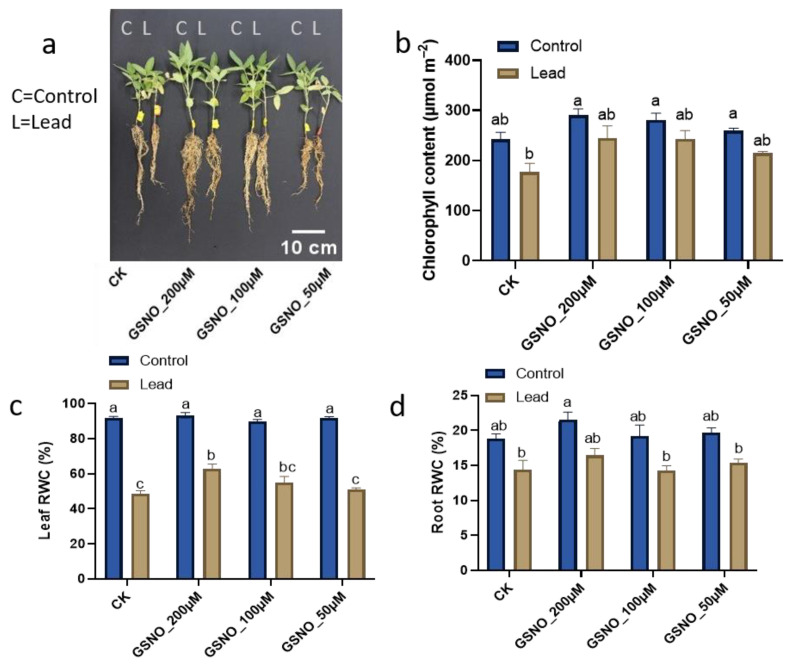
Effect of GSNO supplementation on: (**a**) Phenotype; (**b**) chlorophyll content; (**c**) leaf RWC; (**d**) root RWC, under Pb stress. The mean of three replicates was used. Bars exhibiting different letters indicate significant differences as evaluated by Tukey’s test and Student’s *t*-test at *p* ≤ 0.05.

Our results demonstrated that GSNO has beneficial impacts on soybean seedling growth under Pb stress compared to solely Pb-exposed control plants. (Supplementary Figure S1). Plant and root biomass were significantly reduced under Pb stress. Figure 1b shows that under control conditions, exogenously applied GSNO (200 µM, 100 µM, and 50 µM) increased the chlorophyll content by 19.6%, 15.8%, and 6.9%, respectively, over Pb-unstressed plants. Contrarily, the seedlings exposed to Pb stress alone showed 27.1% less chlorophyll content compared to Pb-unstressed plants, whereas under Pb stress, with 200 µM, 100 µM, and 50 µM NO application, chlorophyll content was increased by 38.7%, 37.8%, and 21.4%, respectively, over Pb-exposed control plants. Under control conditions, there was no significant differences in the relative water contents of leaf and root, except for GSNO_200µM root RWC (Figure 1d), compared to Pb-uninduced control plants. However, under Pb stress, a similar concentration (200 µM) increased the leaf and root RWCs by 29.3% and 14.4%, respectively, compared to the solely Pb-exposed control plants.

### 2.2. Nitric Oxide Supplementation Reduces Lead Content

**Figure 2:**
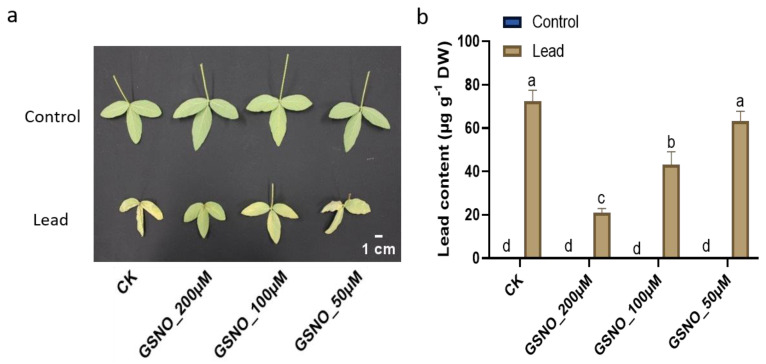
The effect of GSNO supplementation with or without Pb stress on: (**a**) Trifoliate visualization; (**b**) lead content in leaf. The mean of three replicates was used. Bars exhibiting different letters indicate significant differences as evaluated by Tukey’s test and Student’s *t*-test at *p* ≤ 0.05.

In response to Pb stress, a pragmatic accumulation of Pb was noticeable compared to the Pb-unstressed control plants (Figure 2a,b). However, our findings show that, in contrast to lead-treated control plants, GSNO actively reduced the Pb content by 71.1%, 40.3%, and 12.8%, respectively.

### 2.3. Exogenously Applied GSNO Suppresses Lipid Peroxidation, Cell Death, and Oxidative Injury by Altering the Antioxidative Activities under Pb Stress

**Figure 3:**
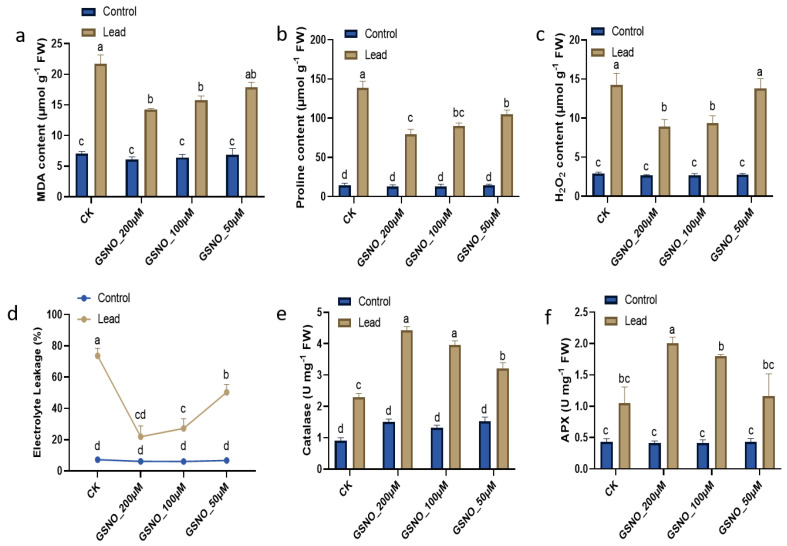
Effect of GSNO supplementation on: (**a**) MDA content; (**b**) proline content; (**c**) H_2_O_2_ content; (**d**) electrolyte leakage; (**e**) catalase activity; (**f**) APX activity under Pb stress. The mean of three replicates was used. Bars exhibiting different letters indicate significant differences as evaluated by Tukey’s test and *t*-test at *p* ≤ 0.05.

Figure 3a shows that Pb stress significantly increased lipid peroxidation and ROS. MDA was significantly increased in the solely lead-stressed plants by three-fold compared to the unstressed control plants. Exogenously applied NO (200 µM, 100 µM, and 50 µM) lessened it by 34.5%, 27.3%, and 17.6%, respectively. Similarly, proline content was dramatically increased to 9.5-fold in the solely lead-stressed plants (Figure 3b) compared to the unstressed control plants. In contrast, the proline content of the plants treated with GSNO (200 µM, 100 µM, and 50 µM) under Pb stress, decreased to 42.5%, 35.3%, and 35.3%, respectively, compared to the solely lead-stressed plants. Furthermore, electrolyte leakage increased 10-fold in the Pb-stressed control plants compared to the unstressed control plants. GSNO reduced the injurious effect by 70.3%, 63%, and 31.7% individually relative to the solely lead-stressed plants (Figure 3d). Similarly, the H_2_O_2_ content was 4.9 times higher in the Pb-stressed plants compared to the unstressed plants (Figure 3c). Contrariwise, NO reduced the H_2_O_2_ content by 37.4%, 33.8%, and 2.8%, respectively, under Pb stress relative to the solely lead-stressed plants.

Furthermore, no significant difference in antioxidant (CAT and APX) activity was found with the GSNO treatments under the Pb-unstressed condition, while exogenous application of NO significantly enhanced the antioxidative activities under the lead-stressed condition (Figure 3e,f). CAT and APX activities were elevated by 58.57% and 60.5%, respectively, compared to the Pb-unstressed condition. However, the exogenous GSNO application at a 200 μM concentration elevated both CAT and APX activities by 1.9-fold each relative to the Pb-treated control plants.

### 2.4. Exogenous GSNO Application Regulates Gene Expression under Lead-Induced Toxicity

**Figure 4:**
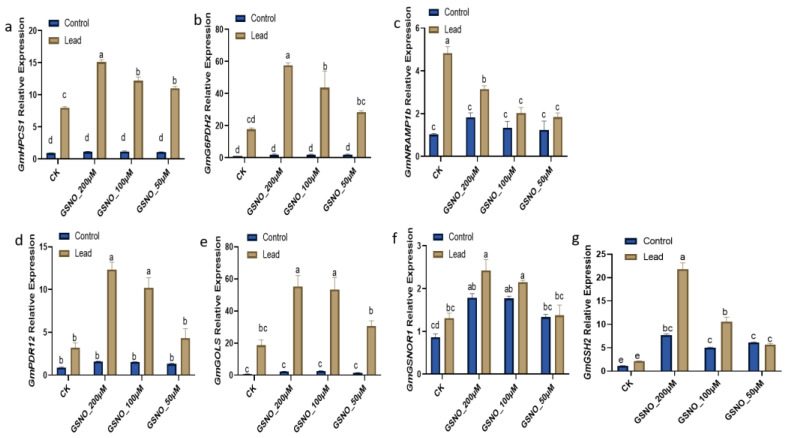
Effect of GSNO supplementation on the relative expressions of: (**a**) *GmHPCS1*; (**b**) *GmG6PDH2*; (**c**) *GmNRAMP1b*; (**d**) *GmPDR12*; (**e**) *GmGOLS*; (**f**) *GmGSNOR1*; (**g**) *GmGSH2*, under Pb stress. The mean of three replicates was used. Bars exhibiting different letters indicate significant differences as evaluated by Tukey’s test and Student’s *t*-test at *p* ≤ 0.05.

To thoroughly investigate the underlying mechanism of nitric oxide-mediated mitigation under Pb stress, the relative expression profiling of stress, metal transporter, and NO-related genes were explored (Figure 4). Pb treatment triggered significantly enhanced gene expressions of the phytochelatin gene *GmHPCS1* (18.5-fold) and the glucose-6-phosphate dehydrogenase gene *GmG6PDH2* (8.7-fold) compared to the Pb-unstressed plants (Figure 4a,b). GSNO supplementation at a 200 μM concentration significantly enhanced the expressions of *GmG6PDH2* and *GmhPCS1* by 225.3% and 89.2%, respectively, compared to the solely lead-treated plants. The expression of heavy metal transporter gene *GmNRAMP1b* significantly increased by 370.3% in the Pb-stressed plants compared to the lead-unstressed plants. However, GSNO supplementation (200 µM, 100 µM, and 50 µM) reduced the relative expressions by 34.7%, 57.8%, and 61.9%, respectively, against lead stress compared to under solely Pb exposure (Figure 4c).

Additionally, the stress-responsive genes *GmPDR12* and *GmGolS* were significantly upregulated under Pb stress compared to the Pb-unstressed plants, whereas GSNO (200 µM, 100 µM, and 50 µM) supplementation greatly enhanced the relative expressions of the *GmPDR12* gene by 283.1%, 217.5%, and 34%, individually and the *GmGolS* gene by 197%, 187.4%, and 64.7%, respectively, compared to under solely Pb exposure (Figure 4d,e).

**Figure 5:**
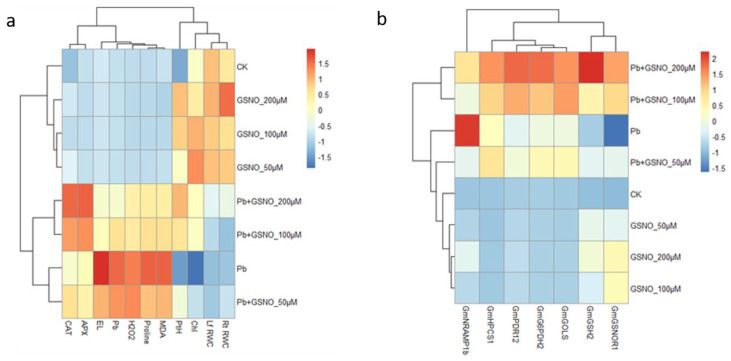
Heatmap showing hierarchical clustering and expression values of different study parameters: (**a**) Physiological and biochemical parameters; (**b**) transcripts in response to treatments. A pheatmap was created in R package using the mean of three replicates where red color expressed positive and blue color expressed negative correlation.

Significant differences were observed in the cases of GSNO-related genes *GmGSH2* and *GmGSNOR1* under the Pb-unstressed condition (Figure 4f,g). *GmGSH2* gene expression was enhanced 6.8-, 4.4-, and 5.4-fold, respectively, and *GmGSNOR1* gene expression was boosted 2.1, 2.0, and 1.5 times, respectively, according to the NO treatment under the Pb-unstressed condition. Interestingly, the relative expression of *GmGSH2* increased (86.8%), but that of *GmGSNOR1* declined (48.5%) in lead-stressed plants compared to the untreated control plants. Under Pb stress, NO application significantly improved gene expression by 10.3, 5.0, and 2.7 times for *GmGSH2* and 5.4, 4.8, and 3.1 times correspondingly for *GmGSNOR1* compared to the Pb-stressed plants. Hierarchical clustering and expression values on physiological, biochemical parameters and relative gene expression in heatmap showing strong positive correlation of electrolyte leakage (Figure 5a) and *GmNRAMP1b* (Figure 5b) both with solo lead exposure.

## 3. Discussion

The present study was conducted to assess the potential of NO to alleviate toxicity induced by Pb in soybean. To the best of our knowledge, this is the first study to investigate the role of GSNO as a donor of nitric oxide to mitigate the toxic effect of Pb on soybean plants. Previously, there were limited reports on other sources of NO donors such as SNP to mitigate heavy metal toxicity [11,13,14]. It has been well reported that lead stress negatively affects plant growth and biomass [5]. We also obtained a similar result, i.e., Pb stress decreased the biomass of soybean; however, NO decreased Pb toxicity in plants (Supplementary Figure S1). Herein, our results demonstrated that exogenously applied NO resulted in better growth under Pb stress, due to the capabilities of NO in sensing, signaling, and stress tolerance in plants under heavy metal stress such as lead stress [18]. NO has been found to improve growth attributes by modulating antioxidative defense systems against heavy metal stress [10]. Several studies have reported that NO also plays an important role in the root system, improving root viability and enriching mineral nutrient acquisition under stress conditions [19]. Chlorophyll pigments in plants are directly related to their photosynthetic capacity and growth [20]. Heavy metal stress has been shown to directly affect chlorophyll pigmentation [21]; however, exogenously applied GSNO increased chlorophyll and relative water content [17]. Our results indicate that NO supplementation helped to reduce the degradation of chlorophyll pigments as well as leaf and root relative water contents according to definite concentrations under Pb stress (Figure 1). Among the treatments, GSNO_200μM was found to have the highest leaf and root relative water contents under Pb-exposed plants and also demonstrated restricting Pb accumulation (Figure 2). This might be due to the membrane integrity protected by the exogenously applied NO under heavy metal stress [14]. Heavy metals directly or indirectly degrade cell membranes and proteins by leading to the overproduction of ROS which is reflected in the MDA level [10]. The results depicted that Pb stress was associated with higher oxidative damage and electrolyte leakage (Figure 3a–d). GSNO supplementation (200 µM and 100 µM concentrations) significantly mitigated the oxidative damage by reducing the accumulation of MDA, proline, and H_2_O_2_. Similar findings have also been reported, with the alleviation of oxidative damage by ROS scavenging using GSNO spraying of plants under stress [17,22]. Consequently, the results revealed that NO has potential to alleviate Pb toxicity and to maintain ion homeostasis in soybean plants. Under Pb exposure, plants usually boost their antioxidative defense mechanisms under stress conditions to mitigate the oxidative damage by scavenging ROS in corn and *Sedum alfredii* [9]. Electrolyte leakage is directly related to cell death and is a hallmark of the stress response in intact plant cells. This phenomenon is widely accepted as a test for stress-induced plant tissue injury and as a measure of plant stress tolerance. Our results demonstrated that Pb stress only and low concentrations (50 µM) of GNSO caused more electrolyte leakage in Pb-induced plants, which in turn caused more cell death in soybean leaves (Figure 3d). These results suggest that NO enhances the ability of plants to uptake nutrients that trigger better growth and less membrane damage against heavy metal stress. Moreover, our outcomes indicate that exogenously applied GSNO increases the activity of antioxidants CAT and APX (Figure 3e,f) which might help to scavenge ROS to produce water molecules. Our results further confirmed the findings in heavy metal-stressed soybean, wheat, and tomato plants [10,14,23]. Taken together, antioxidants were boosted profoundly, which were assigned to the mitigation behavior of GSNO against lead-induced oxidative damage in soybean plants. Exogenously applied GSNO induced less Pb absorption and accumulation in soybean plants, which might be related to the responsive genes in terms of ROS, stress, and metal transport. Furthermore, phytochelatins are a significant class of heavy metal chelators, which are a common means of metal detoxification present in a variety of plants [24]. Several studies have demonstrated that PCS genes have been upregulated under chromium exposure in soybean [10], lead exposure in *Medicago* and *Arabidopsis* [25,26], and cadmium exposure in tomato [26]. Our results also depicted an enhanced expression of the *GmhPCS1* gene under Pb exposure, which was elevated with GSNO supplementation (Figure 4a). Recently, *G6PDH* isoform genes have gained considerable attention as they play an important role against environmental stress through the oxidative pentose pathway [27]. *GmG6PDH* has been reported to be upregulated in soybean under aluminum stress [28]. Here, our results indicated that *GmG6PDH2* was upregulated significantly under lead exposure, while gene expression was enhanced more with GSNO application (Figure 4b), which probably worked for NADPH biosynthesis during Pb translocation. Improved relative expression of the metal transporter gene GmNRAMP1b suggests higher accumulation of metal binding under solely Pb exposure compared to other treatments (Figure 4c). *GmNRAMP* genes have been well studied against diverse heavy metals such as cadmium, iron, and copper toxicity and have been found to be responsible for tolerance to metals in plants [29]. *AtGolS1* increased plant growth in *Arabidopsis* under arsenic toxicity [30]. A higher content of galactinol synthase was reported in rice under Pb stress [31]. Likewise, we observed upregulation of the *GmGolS1* gene under Pb exposure, which was enhanced with GSNO supplementation. Additionally, GmPDR12 gene expression, which is an ABC transporter gene, was significantly enhanced under Pb exposure with GSNO supplementation (Figure 4d). Similar findings were reported in *Arabidopsis* under Pb exposure [32] where excess Pb(II) containing toxic compounds were found to pump out from cytosol. Since Pb tolerance is related to the ability of plants to restrict Pb to the cell walls, regulate osmolytes, and their antioxidant defense systems; thus, the correct concentration of exogenous NO supplementation contributes to plant growth and detoxification of Pb-induced stress. Further, we checked the relative expression of NO-related genes, *GmGSNOR1* and *GmGSH2*; both of the gene expressions were increased under Pb exposure, however, with the addition of GSNO, they were found to be further increased compared to under solely Pb exposure. Under Pb stress, the *GSH2* gene is directly related in GSH production which is converted to phytochelatin in the presence of phytochelatin synthase, and thus, helps in Pb detoxification in higher plants [33]. The aldehyde dehydrogenase class-3 gene *GmGSNOR* has been shown to be upregulated under Pb and cadmium stress with exogenous NO application in soybean [11]. However, the glutathione synthase genes *GmGSH1* and *GmGSH2* genes have been found to be significantly higher in roots than leaves in soybean under chromium toxicity with GSH supplementation [10]. In summary, the upregulation of NO, PCS, and metal transport genes with GSNO application validates the detoxification of ROS induced by the toxic metal Pb in soybean.

## 4. Materials and Methods

### 4.1. Plant Material and Growth Condition

The seeds of the Korean elite cultivar, Pungsannamul, were donated by the Soybean Genetic Resource Center (Kyungpook National University, Daegu, Republic of Korea). The surface sterilization of seeds was performed with 70% ethanol, followed by washing five times with autoclaved distilled water, and then the seeds were immediately sown in pots. Each pot was filled with 1 kg of sandy soil without the addition of any fertilizer. Pots were placed in the greenhouse of Kyungpook National University, South Korea, with a controlled photoperiod (16 h light/8 h dark cycle) and temperature (28 °C ± 2 °C). The experiment was conducted in a complete randomized design with three replications. At the V3 stage (first fully expanded trifoliate), plants were randomly allocated into the following eight groups: only water, lead, GSNO_50µM, lead + GSNO_50µM, GSNO_100µM, lead + GSNO_100µM, GSNO_200µM, and lead + GSNO200 µM. Since the soybean is moderately tolerant to lead stress, plants were treated twice (1st day and 3rd day) with lead nitrate (Sigma-Aldrich, St. Louis, MI, USA) by soil drenching at a higher concentration (1 mM) for lead application. Sodium nitrite (NaNO_2_) was added in an equimolar ratio to nitrosylate-reduced glutathione (GSH), and GSH was dissolved in hydrochloric acid (1N) for the preparation of GSNO.

### 4.2. Chlorophyll Content and Relative Water Content

Chlorophyll content was measured using an Apogee chlorophyll concentration meter (MC-100, Apogee Instruments Inc., Logan, UT, USA) and expressed in mol of chlorophyll per m^2^. The relative water content (RWC) of leaf and root was measured following the method described by Valentovic et al. [34]. Briefly, leaf and root samples were collected and washed gently with water three times. Fresh weight (FW) was recorded, and after hydrating the samples for 2 h, turgor weight was determined. Then, the samples were dried at 65 °C for at least 48 h and dry weight was recorded. RWC was calculated using the following formula:

$$\mathrm{RWC}\left(\%\right)=\frac{\left(\mathrm{FW}-\mathrm{DW}\right)}{\left(\mathrm{TW}-\mathrm{DW}\right)}\times 100$$

### 4.3. Lipid Peroxidation, Proline, and H_2_O_2_ Content

Lipid peroxidation was measured in terms of malondialdehyde (MDA) content [35]. Briefly, leaf samples were homogenized with 5% thiobutyric acid and analyzed with 20% trichloroacetic acid. The mixture was incubated for 25 min at 95 °C in a water bath, followed by cooling down in ice and centrifugation again to clarify the solution. Absorbance was measured at 532 nm and 600 nm with a spectrophotometer (Shimadzu UV-1280, Kyoto, Japan). The MDA level was calculated with an extinction coefficient of 155 mM^−1^ cm^−1^ and expressed as micromoles of MDA g^−1^ FW. Proline content was estimated according to method reported by Bates et al. [36]. In brief, the samples were digested in 3% sulfosalicylic acid, and an equal amount of supernatant was taken to react with acid ninhydrin and glacial acetic acid. The solution was incubated at 100 °C for 1 h. The reaction was terminated in an ice bath. Toluene (4 mL) was added in the tubes, and the aqueous phase of the aspirated solution was taken to record the absorbance at 520 nm using toluene as a blank and expressed as micromoles per gram FW. The quantification of H_2_O_2_ was performed after reaction with potassium iodide, as described by Alexieva et al. [37]. Leaf samples were homogenized with trichloroacetic acid (1%). Leaf extract supernatant, potassium phosphate buffer, and potassium iodide reagents were mixed and incubated in the dark for 1 h. The absorbance was measured using a spectrophotometer at 390 nm. The quantities of proline and H_2_O_2_ were calculated using a standard curve.

### 4.4. Electrolyte Leakage, Catalase, and Ascorbate Peroxidase Content

Electrolyte leakage (EL) was measured as described by [34] with a slight modification. Briefly, 1 cm diameter of two leaf disks of same plant with three replicates for each treatment were collected and washed vigorously with distilled water. Later, the disks were placed in a tube with 10 mL of deionized water, which was incubated for 2 h at room temperature. The electrical conductivity (L1) of the solution was recorded using a portable conductivity meter (HURIBA Twin Cond B-173, Japan). Then, the samples were autoclaved at 120 °C for 20 min, and the final conductivity (L2) was measured soon after equilibration at 25 °C. EL was calculated using the following formula:

$$\mathrm{EL}\left(\%\right)=\frac{L1}{L2}\times 100$$

Activity of catalase (CAT) was conducted following [38], and activity of ascorbate peroxidase (APX) was conducted following [39]. Briefly, for catalase activity, 75 mM H_2_O_2_, 0.1 M phosphate buffer, and 50 L of diluted enzyme extract were used and calculated with an extinction coefficient of 39.4 mM^−1^ cm^−1^ at 240 nm absorbance. APX activity was observed at an absorbance of 290 nm with an extinction coefficient of 2.8 mM^−1^ cm^−1^. The assay mixture consisted of enzyme extract, 0.1 M EDTA, 50 mM phosphate buffer, 0.5 M ascorbate, and 1 mM H_2_O_2_. The Coomassie protein assay kit (Thermo Fisher Scientific, Waltham, MA, USA) was used to determine the total protein content with the Bradford assay method [40], following the manufacturer’s manual.

### 4.5. Lead Content

Lead content was estimated using ICP-MS (Optima 7900DV, PerkinElmer, Waltham, MA, USA). Briefly, the sample was homogenized with nitric acid (HNO_3_, 5 mL) and H_2_O_2_ (3 mL), and the diluted (3% HNO_3_) supernatant was filtered through a 0.45 μm syringe filter (Sterlitech, Auburn, WA, USA) and then injected into a coupled plasma mass spectrometry analyzer.

### 4.6. Relative Gene Expression Analysis

Quantitative real-time PCR (qRT-PCR) was performed to analyze the gene expression of phytochelatin (*hPCS1*), glucose-6-phosphate dehydrogenase (*G6PDH2*), metal transporter-related (*NRAMP1b*), ATP binding cassette-related (*PDR12*), galactinol synthase (*GolS1*), and nitric oxide related (*GSH2* and *GSNOR1*). Soybean leaf samples were ground with liquid nitrogen, and RNA was extracted using the TRIZOL reagent. RNA concentration and purity were checked using NanoQ (Korea). A cDNA synthesis kit (BioFACT^TM^ RT-Kit) was used to synthesize cDNA, following the manufacturer’s instructions. Later, cDNA was used as a template to assess relative gene expression for the qRT-PCR Eco^TM^ real-time PCR machine (Illumina, San Diego, CA, USA) using BioFACT^TM^ 2X real-time PCR Master Mix (including SYBR^®^ Green I). *GmELF1b* was used as the reference gene. The gene expression was performed in triplicate. A detailed primer list of corresponding genes is provided in the Supplementary Materials, Table S1.

### 4.7. Statistical Analysis

All the experiments were performed with three replications, and mean values were compared by Tukey’s test using SAS version 9.4 (SAS Institute Inc., Cary, NC, USA) software and Student’s *t*-test, where *p* ≤ 0.05. GraphPad Prism (San Diego, CA, USA) version 9.0.0 (121) was used for the graphical presentation of results. A pheatmap was created using R package (version 4.2.2) [41].

## 5. Conclusions

Heavy metals cause a reduction in crop productivity by damaging plant growth and development. Pb adversely affected plant growth and development via the excessive production of ROS. NO has been reported as a signaling molecule and is also known to be a main factor in maintaining homeostasis at the cellular level in plants. In this study, we investigated the role of NO as a mitigator of the toxic effect of lead on soybean plants by applying different doses of GSNO. According to our results, GSNO supplementation (200 µM and 100 µM) reduced oxidative damage. Moreover, GSNO application resulted in increased chlorophyll content and positively modulated the enzymatic antioxidant activities to lessen ROS and lead detoxification. Therefore, our results indicated that NO has a potential role in the lead tolerance adaptive response mechanism in soybean.

## Data Availability

Data would be made available upon request.

## Supplementary Materials

The supporting information can be downloaded at: https://www.mdpi.com/article/10.3390/ijms24129901/s1.
