# Supplementary material for: S-Nitrosoglutathione (GSNO)-Mediated Lead Detoxification in Soybean through the Regulation of ROS and Metal-Related Transcripts
Source: Int J Mol Sci. 2023 Jun 8;24(12):9901. doi: 10.3390/ijms24129901 (PMC10298733; doi:10.3390/ijms24129901)
Supplement: Supplementary file 1 [file ijms-24-09901-s001.zip › ijms-2391401-supplementary.pdf]

Table S1. List of primers used for real-time PCR analysis.

	Gene Name	Description	Forward Primer	Reverse Primer
**1.**	*GmhPCS1*	Phytochelatin synthase 1 (PCS1)	AAGGCTTGCTATCAAGGAAGAGG	CTGATACTTGTTGCCTGCTGC
	GmG6PDH2	Glucose-6-phosphate dehydrogenase	AGCTAACTCTGCTTCACTTGGT	GCTGGTAAGAATCCCTGCCG
**2.**	*GmNRAMP1b*	Metal transporter NRAMP2-related	GAATTGGCCCTGTCCTCAAGATTA	GTGGTAGCAACTGTCTTTGATCG
**3.**	GmPDR12	Pleiotopic drug resistant / ABC transporter	TATGCAGTGTGGAATCTCTTCTCAG	CAATCCATACAAACTCCATGCTACA
**4.**	GmGolS	Galactinol synthase	GTGACCACAACCCTTCCTCC	TCAACGTTCTCAGGGTGACG
**5.**	*GmGSNOR1*	S-Nitrosoglutathione Reductase / alcohol dehydrogenase class-3	TGTTGGGTTGCTTTTCCCCT	TCTTTCTGATCACAAGTTTGCCA
**6.**	*GmGSH2*	Glutathione synthase / GSH synthetase	GAAAGAGGCCAAAATGGGCG	ACGCTTCCTGATCTCTGAACG
**7.**	*GmELF1b*	*Reference gene*	CCACTGCTGAAGAAGATGATGATG	AAGGACAGAAGACTTGCCACTC

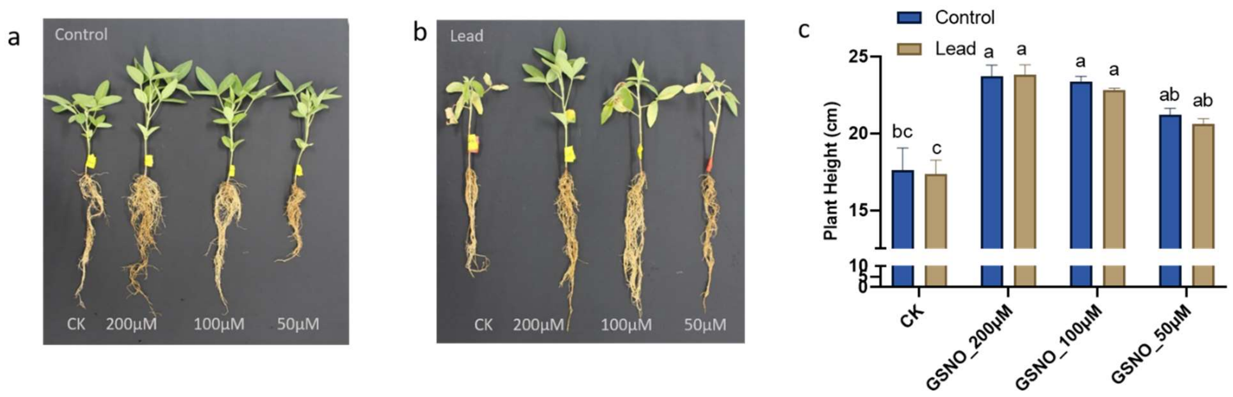

Figure S1. Effect of GSNO supplementation on (a) phenotype under control condition, (b) phenotype under lead stress and (c) plant height under lead stress. The mean of three replicates were used. Bars exhibiting different letters indicate significant differences as evaluated by Tukey's test and t-test at P ≤0.05.
